# Essential Oil from *Zingiber ottensii* Induces Human Cervical Cancer Cell Apoptosis and Inhibits MAPK and PI3K/AKT Signaling Cascades

**DOI:** 10.3390/plants10071419

**Published:** 2021-07-12

**Authors:** Jirapak Ruttanapattanakul, Nitwara Wikan, Kittinan Chinda, Thanathorn Jearanaikulvanich, Napatsorn Krisanuruks, Muantep Muangcha, Siriporn Okonogi, Saranyapin Potikanond, Wutigri Nimlamool

**Affiliations:** 1 Department of Pharmacology, Faculty of Medicine, Chiang Mai University, Chiang Mai 50200, Thailand; jirapak.ken@gmail.com (J.R.); kittinan_chinda@cmu.ac.th (K.C.); thanathorn_j@cmu.ac.th (T.J.); napatsorn_kr@cmu.ac.th (N.K.); muantep_muangcha@cmu.ac.th (M.M.); saranyapin.p@cmu.ac.th (S.P.); 2 Institute of Molecular Biosciences, Mahidol University, Nakorn Pathom 73170, Thailand; nitwara.wik@mahidol.edu; 3 Department of Pharmaceutical Sciences, Faculty of Pharmacy, Chiang Mai University, Chiang Mai 50200, Thailand; siriporn.okonogi@cmu.ac.th; 4 Research Center of Pharmaceutical Nanotechnology, Faculty of Pharmacy, Chiang Mai University, Chiang Mai 50200, Thailand

**Keywords:** *Zingiber ottensii*, essential oil, anti-cancer, apoptosis, cervical cancer, IL-6

## Abstract

*Zingiber ottensii* (ZO) is a local plant in Thailand and has been used as a Thai traditional therapy for many conditions. ZO has been reported to exhibit many pharmacological effects, including anti-cancer activity. Nevertheless, its anti-cancer effects explored at the signaling level have not been elucidated in cervical cancer, which is one of the leading causes of fatality in females. We discovered that the essential oil of ZO significantly increased the apoptosis of human cervical cancer cells (HeLa) after 24 h of treatment in a concentration-dependent manner. Our data also clearly demonstrated that ZO essential oil reduced IL-6 levels in the culture supernatants of the cancer cells. Moreover, Western blot analysis clearly verified that cells were induced to undergo apoptotic death via caspase activation upon treatment with ZO essential oil. Interestingly, immunofluorescence studies and Western blot analyses showed that ZO essential oil suppressed epidermal growth factor (EGF)-induced pAkt and pERK1/2 signaling pathway activation. Together, our study demonstrates that ZO essential oil can reduce the proliferation and survival signaling of HeLa cervical cancer cells. Our study provides convincing data that ZO essential oil suppresses the growth and survival of cervical cancer cells, and it may be a potential choice for developing an anti-cancer agent for treating certain cervical cancers.

## 1. Introduction

Cancer is a defect of the dysregulation of cell growth and cell invasion to nearby cells or different cells of the body [1]. Hyperplasia that is not invasive or metastatic is called “benign” but is not “cancer.” One of the most diagnosed cancers in females around the world is cervical cancer [2]. The main cause for cervical cancer is from the infection of the cervical epithelia caused by high-risk human papilloma viruses (HPVs). HPV-18 and HPV-16 play important roles in causing most cervical cancers [3]. The symptom of cervical cancer can be divided into two stages; the first one is early-stage cervical cancer that typically produces no signs or symptoms, and the second one is more-advanced cervical cancer that can develop clinical symptoms including watery and bloody vaginal discharge, vagina bleeding, and pelvic pain [4]. Currently, the common line of treatment includes chemotherapy, radiation, and tumor surgery; these therapies can be applied as monotherapy or in combination. However, most of these treatment choices often cause side effects to patients. For example, certain chemotherapeutic drugs can cause bone marrow suppression, gastrointestinal disturbance, nausea, vomiting, and pancytopenia, etc., which could significantly decrease the quality of life of patients [5,6]. Therefore, new forms of therapy should be discovered to eliminate the unwanted side effects while providing effective curing benefits to patients with cervical cancer.

Many plants with known biological activities have long been used in Thailand as traditional remedies for treating various diseases [7,8,9]. Specifically, several plants in the Zingiberaceae family are typically used in Thailand, and their various pharmacological effects have recently been explored. For example, *Curcuma amarissima* or “black turmeric” and *Boesenbergia rotunda* or “Fingerroot” have been reported for their wound-healing-promoting activities [10,11], while *Kaempferia parviflora* or “Thai ginseng” is now known for its strong anti-cancer activity [12].

*Zingiber ottensii* (ZO) is also a plant in the Zingiberaceae family [13]. This plant is found in Thailand and has been used as traditional medicines to treat various symptoms. *Zingiber ottensii* is called “Plai-dum” in Thai. Several pharmacological studies of *Zingiber ottensii* have claimed the benefits of this plant for various ailments. However, its toxicity to certain organisms has also been demonstrated. For example, the essential oil of ZO has been shown to cause toxicity and the aberrant embryogenesis of zebrafish [14]. Regarding its cellular toxicity, there was a report showing that ZO induced death in some cancer cell lines [15]. In association with its cytotoxicity, phytochemical analysis revealed that *Zingiber ottensii* contains the large amount of sesquiterpenes, with zerumbone as its major constituent [16]. In an in vitro study, zerumbone was examined for its inhibitory activities against tumor-promoting cytokine production [17]. Nevertheless, pharmacological research related to the anti-cervical cancer activity of *Zingiber ottensii* has not been elucidated, especially at the molecular signaling level. Therefore, we aimed to investigate the anti-cancer activities of *Zingiber ottensii* (ZO) against human cervical cancer cells by focusing on its ability to induce cervical cancer cell apoptosis, cell survival, proliferation, and relevant signal transduction pathways. Our current study provides accumulated information that *Zingiber ottensii* can induce cervical cancer cell death through the caspase-dependent pathway, and this effect is associated with its ability to reduce ERK1/2 and Akt activation.

## 2. Results

### 2.1. ZO Essential Oil Stimulates Apoptosis in HeLa Cells

**Figure 1:**
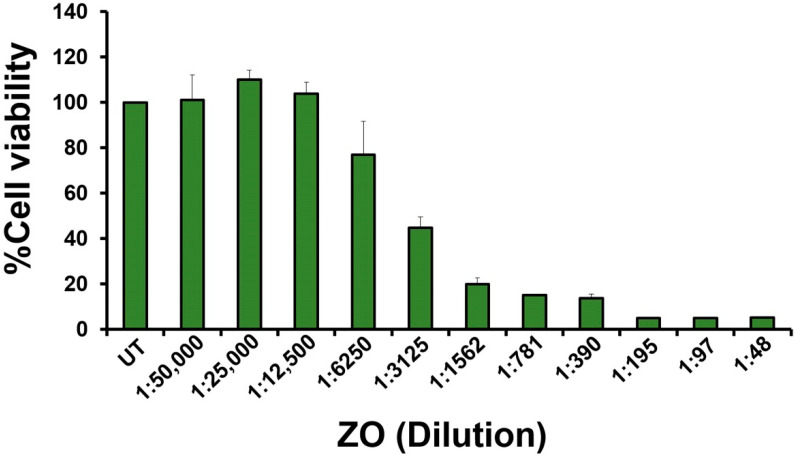
The effect of ZO essential oil on HeLa cell viability. The viability of HeLa cervical cancer cells upon being treated with ZO essential oil for 24 h was tested by an MTT assay. The treatment concentrations of ZO essential oil were prepared by performing a 2-fold dilution before addition to the cells. The experiment was performed three times (triplicate each time).

After obtaining the *Zingiber ottensii* (ZO) essential oil, its cytotoxic effects on HeLa cervical cancer cells were evaluated by an MTT assay. HeLa cells were treated with ZO essential oil at various dilutions ranging from 1:50,000 to 1:48 (prepared by a two-fold dilution method) for 24 h. The results from the MTT assay clearly showed that ZO essential oil strongly decreased the viability of HeLa cells when the concentration was increased. Specifically, ZO essential oil at the dilutions of 1:12,500, 1:25,000, and 1:50,000 was not toxic to the cell, as it can be seen in the results that the viability of HeLa cells was maintained to be around 100% when compared to the untreated group (Figure 1). However, ZO essential oil at the dilution of 1:6250 started to reduce the viability of HeLa cells to approximately 80%. Moreover, ZO essential oil at around 1:3000 was seen to be the concentration that caused a 50% reduction of HeLa cell viability, and ZO essential oil at higher concentrations (dilutions of 1:1562 to 1:48) caused a maximal reduction of cell viability (Figure 1). In addition, we tested the cytotoxic effects of ZO essential oil on the cell viability of normal cells, which were human primary fibroblast cells. As shown in Supplementary Figure S1, we found that the IC50 of ZO essential oil for human primary fibroblast cells was approximately 1:390, and the 1:3000 and 1:6000 dilutions of ZO essential oil had no cytotoxic effects on human primary fibroblast cells. Consistently, when we monitored the morphology of human primary fibroblast cells treated with 1:3000 and 1:6000 of ZO essential oil, we observed no significant differences between the untreated group and the ZO-treated groups (Supplementary Figure S2). Specifically, HeLa cervical cells treated with ZO essential oil at 1:3000 and 1:6000 did not exhibit apoptotic characteristics but maintained the morphology of healthy fibroblast cells. These data indicate that HeLa cervical cancer cells are more sensitive to ZO essential oil treatment than human primary fibroblast cells. Since we mainly aimed to determine the effects of ZO essential oil on inducing cervical cancer cell death, we selected the concentration of ZO essential oil at 1:6000 and 1:3000 (IC50) dilutions to evaluate its ability to induce HeLa cervical cancer cell death.

**Figure 2:**
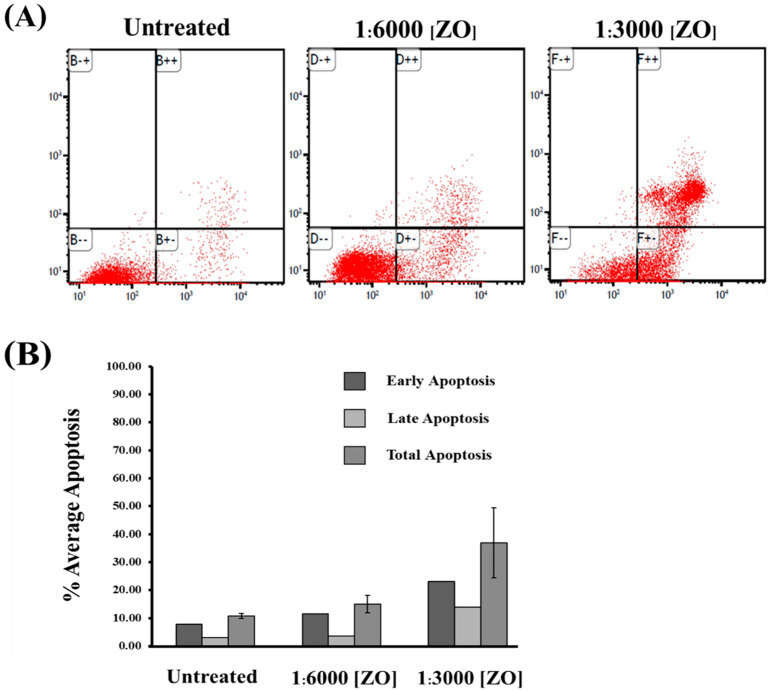
The effect of ZO essential oil on HeLa cell apoptosis. (**A**) A representative histogram for flow cytometry demonstrating HeLa cell apoptosis after 24 h of treatment with different dilutions (1:6000 and 1:3000) of ZO essential oil and stained with annexin V/PI. (**B**) Quantitative analysis representing the average percentage of apoptotic HeLa cells at the early, late apoptosis, and total apoptosis in HeLa cells treated with 1:6000 or 1:3000 dilutions of ZO essential oil for 24 h.

Next, we performed apoptosis analysis by staining the cells with annexin V/PI and detected the percent of apoptotic cells by using flow cytometry. The results demonstrated that the amount of apoptotic HeLa cells including early, late, and total apoptotic cells after treatment with ZO essential oil for 24 h significantly increased in a dilution-dependent fashion. In particular, the amount of early apoptotic HeLa cells was elevated to approximately 10% and 20% in HeLa cells treated with 1:6000 and 1:3000 dilutions of ZO essential oil, respectively (Figure 2A,B). Similarly, when the total cell death was calculated, it showed that ZO essential oil at the 1:6000 and 1:3000 dilutions clearly contributed to approximately 15% and 35% apoptotic death, respectively, in HeLa cells (Figure 2B).

### 2.2. Effects of ZO Essential Oil on Decreasing IL-6 Production in HeLa Cells

**Figure 3:**
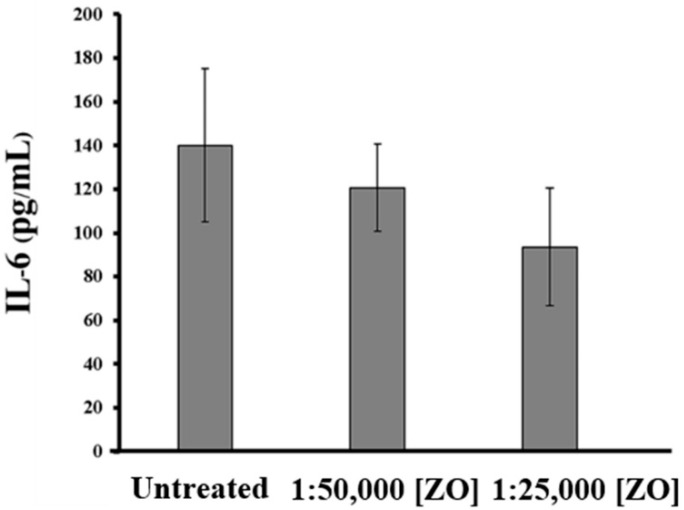
Measurement of IL-6 (pg/mL) in the culture media of HeLa cells incubated with various dilutions of ZO essential oil for 24 h by ELISA.

To investigate whether ZO essential oil can suppress the production of interleukin 6 (IL-6), which is one of the specific cytokine markers in HeLa cells responsible for cervical cancer development and progression [18,19,20,21,22], ELISA was performed to measure the amount of IL-6 in the culture media of HeLa cells treated with ZO essential oil at different dilutions for 24 h. The results demonstrated that the IL-6 levels in the culture supernatants decreased in when the dilution of ZO essential oil was increased. Specifically, the amount of IL-6 in the media of HeLa cells without any treatment was detected to be approximately 140 pg/mL, but ZO essential oil at dilutions of 1:50,000 and 1:25,000 (the non-cytotoxic concentrations) reduced the production and secretion of IL-6 into the culture media to approximately 120 and 90 pg/mL, respectively (Figure 3). These data strongly suggest that ZO essential oil may suppress IL-6-mediated cancer cell progression, at least through inhibiting the production and secretion of IL-6.

### 2.3. Effects of ZO Essential Oil on Inducing Apoptosis Signaling in HeLa Cells

**Figure 4:**
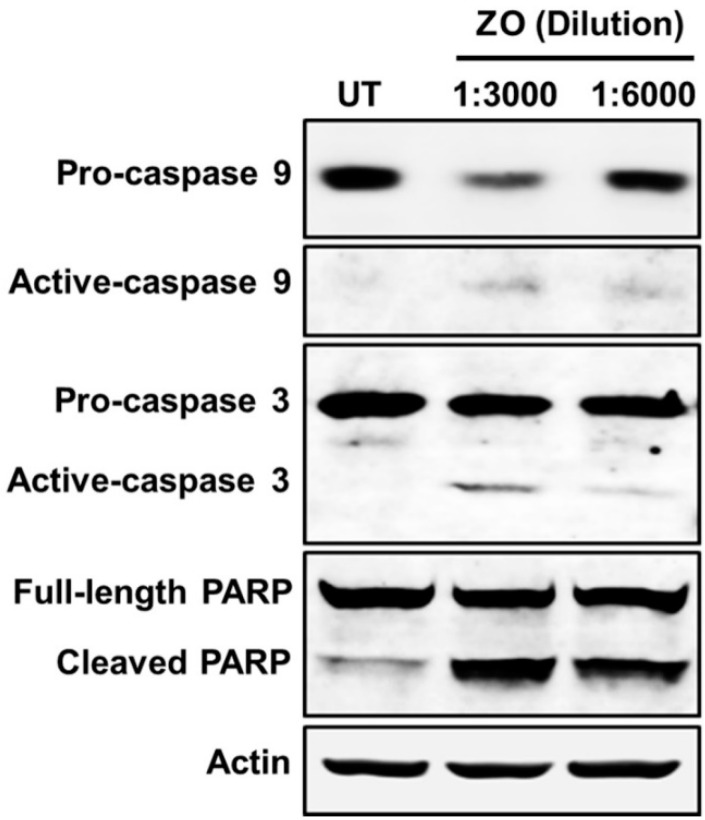
Western blot analysis detecting apoptosis signaling markers, including caspase 9, caspase 3, and PARP in HeLa cells treated with ZO essential oil at 1:3000 and 1:6000 dilutions for 24 h.

We performed Western blot analysis to investigate molecular markers relevant to apoptotic cell death in HeLa cell lysates. The results demonstrated that HeLa cells treated with ZO essential oil illustrated a significant reduction of pro-caspase 9, pro-caspase 3, and full-length PARP protein, whereas the elevation of active caspase-9, active caspase-3, and cleaved PARP protein was increased (Figure 4). These data verified that ZO essential oil truly induced HeLa cell death via apoptosis signaling.

### 2.4. Effects of ZO Essential Oil on the Growth and Survival Signalings in HeLa Cells

**Figure 5:**
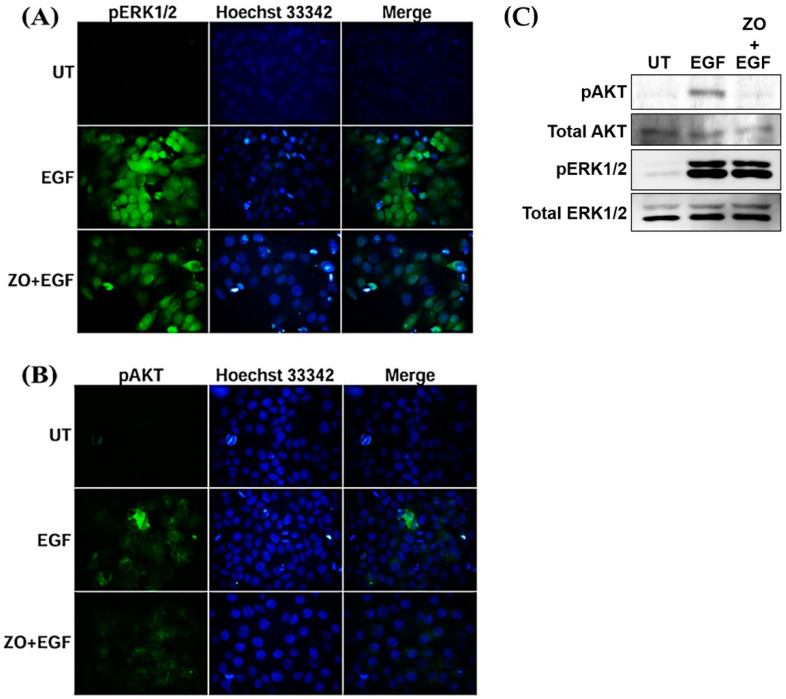
Immunofluorescence study for the effects of ZO essential oil at the dilution of 1:25,000 on (**A**) pERK1/2 and (**B**) pAkt activation. HeLa cells were stained with Hoechst 33342 for nuclear staining (blue). Micrographs were photographed by a fluorescent microscope (100× magnification). (**C**) Western blot analysis detecting the phosphorylation (activation) status of ERK1/2 and Akt kinases and their total protein expression.

We performed an immunofluorescence assay to detect the alteration of cell proliferation and survival molecular markers after EGF stimulation. After stimulating with EGF, the intensity of pERK1/2 and pAkt was detected in comparison to the untreated group, which presented a negative signal (Figure 5A,B). However, when EGF-treated HeLa cells were cultured with the presence of ZO essential oil, we observed a slight reduction of the phosphorylation of ERK1/2 (Figure 5A) and a drastic decrease in Akt phosphorylation (Figure 5B). Consistently, the results from Western blot analysis clearly verified that the activation (phosphorylation) of ERK1/2 in response to EGF stimulation was slightly reduced when ZO essential oil was present (Figure 5C). However, ZO essential oil was very potent in inhibiting the EGF-stimulated phosphorylation of Akt kinase, which is a crucial kinase to maintain cervical cancer cell survival (Figure 5C). These data suggest that ZO essential oil at non-toxic dilutions can also suppress the EGF-mediated growth and survival signal transduction pathways in HeLa cervical cancer cells.

## 3. Discussion

It is well known that a noninfectious disease called “cervical cancer” is categorized to be one of the most common causes of death in the female population around the world [2]. Furthermore, screening programs for cervical cancer are not affordable for everyone, and treatments are not effective and also expensive [6]. Therefore, the discovery of new effective and affordable medicines is necessary. In Thailand, many plants are suitable to be developed as an alternative medicine because they possess certain pharmacological activities and have been used to treat different conditions or diseases [23,24,25,26]. *Zingiber ottensii* (ZO) or Plai-dum is an herb in the Zingiberaceae family found to be prevalent in Thailand [13]. The rhizomes are used as a spice in local dishes. The plant is used in traditional medicine. Specially, the essential oil of this plant is the main ingredient in its ointment that is effective against a wide range of conditions, including cuts, bruises, sprains, ulcers, and muscle pain. Moreover, the oil can be used to alleviate skin rash and inflammation. These anti-inflammatory effects of ZO essential oil were examined in macrophage cell line [27]. The hydrodistilled essential oil from the rhizome of ZO was explored to contain several different constituents, with zerumbone (24.73%) identified to be the major component that had high toxicity [14]. Furthermore, it was shown that the pupae of *Aedes aegypti* and *Culex quinquefasciatus* mosquitoes were susceptible to ZO essential oil [28]. For an aspect of anti-cancer properties, it has reported that Zingipain, which is a cysteine protease isolated and purified from *Zingiber ottensii* rhizomes, possessed relatively strong anti-proliferative effects against cancer cell lines including hepatocellular carcinoma, HEP-G2 (IC50 = 1.13 µg/mL), and colon cancer, SW620 (IC50 = 5.37 µg/mL) [15]. However, there had previously been no investigation for the anti-cancer activities of the essential oil from *Zingiber ottensii* at the signaling pathway level to explain its molecular mechanisms of action. As a step of drug discovery and development, an attempt to identify specific target molecules that have effectiveness in curing cancers is necessary. Therefore, we explored *Zingiber ottensii* essential oil for its anti-cancer activities by using human cervical cancer cell line (HeLa) as a study model. We performed flow cytometry analysis to evaluate apoptotic cell population after treatment with ZO essential oil by using an annexin V and PI staining assay. Interestingly, the results demonstrated that the number of apoptotic HeLa cells after treatment with ZO essential oil for 24 h increased in a dilution-dependent manner in both early and late apoptosis. The phenomenon that caused the population of HeLa cells to shift to the apoptotic area in both early and late stages after being treated with ZO essential oil may be caused by the activation of intrinsic apoptotic signaling via the activation of executive caspases and the PARP protein. To prove this possibility, we performed Western blot analysis to monitor caspase activation. As anticipated, the results from the Western blot analysis clearly demonstrated that ZO essential oil could decrease the band intensity of different pro-caspases and the PARP protein, as well as increase the immunoreactive bands of cleaved caspase and cleaved PARP, indicating the activation of the intrinsic apoptotic pathway. Moreover, we performed immunofluorescence staining and Western blot analysis to investigate the proliferation and survival signaling pathways of HeLa cells after treatment with ZO essential oil and stimulated with EGF. Interestingly, immunofluorescence study results clearly demonstrated that after EGF stimulation, pERK1/2 and pAkt intensity were strongly increased when compared to the untreated group. However, EGF-treated cells with the presence of ZO essential oil showed reductions of ERK1/2 and Akt phosphorylation. Undoubtedly, an effective decrease in the post translational modification of ERK and Akt resulted in the reduction of cancer cell proliferation and survival after treatment with ZO essential oil. Nevertheless, how ZO essential oil specifically interferes with the upstream growth and survival signaling pathways is not known. Addressing this question is quite challenging since the complexity of the kinase–substrate interaction is not fully defined and may require research areas that conduct the rational redesign of a functional kinase–substrate interaction to accumulate the information related to cellular consequences regulated by a specific kinase and target pair [29].

We further explored inflammatory cytokine production in the culture supernatants of HeLa cells treated with ZO essential oil. We mainly focused on IL-6, which has been demonstrated to be one of the key players for the development and progression of cervical cancer cells with HPV infection. Specifically, it is known that HPV16/18 infection governs cells to produce E6 proteins, which consequently activate the IL-6/STAT3 signaling responsible for the reorganization of the microenvironment around tumor that enhances the transformation of the epithelial cells from a chronic tumor-prone inflammatory state to a malignant proliferative state [22]. Therefore, ELISA was performed to evaluate effect of ZO essential oil on IL-6 production. The results clearly demonstrated that the amount of IL-6 decreased in a dilution-dependent manner. The reduction of IL-6 by ZO essential oil suggests that ZO essential oil may help suppress the production of this inflammatory cytokine which is involved in tumorigenesis [18].

Recently, we reported that ZO essential oil isolated from *Zingiber ottensii* Valeton contains several different potential compositions (assayed by the GC–MS method), including zerumbone (24%), terpinen-4-ol (18%), sabinene (15%), and β-pinene (7%) [14]. In particular, zerumbone is an interesting natural crystalline cyclic sesquiterpene because it has been stated to have application potential in chemoprevention and chemotherapy approaches, both in vitro and in vivo [30]. Furthermore, strong evidence from many studies has demonstrated that zerumbone effectively suppresses the proliferation of various cancer types including breast cancer, colon cancer, liver cancer, and cervical cancer, and it has also been shown to have better selectivity on cancer cells compared to healthy cells [31,32,33]. On this basis, our study suggests that ZO essential oil possesses anti-cancer activities that include the induction of apoptotic signaling pathway and the suppression of growth and survival signaling pathways in human cervical cancer cells. This study sheds light on the possibility to develop an anti-cancer agent for preventing or curing cervical cancer.

## 4. Materials and Methods

### 4.1. Plant Material and Isolation of Zingiber ottensii Essential Oil

*Zingiber ottensii* (ZO) rhizomes were collected from the local farm located in Mhae Thang, Chiang Mai province, Thailand. The identification of *Zingiber ottensii*, with the voucher specimen (000109), was performed, and the plant samples were deposited at Chiang Mai University (Faculty of Pharmacy). For extracting the oil, rhizomes of the plant were thoroughly minced. The essential oil was isolated by performing steam distillation. First, the plant was put in a steaming supply. The rhizomes were exposed to water steam at 100 °C. The essential oil was carried by the steam to a condenser section, which then separated in a separator. The ZO essential oil was kept in the dark at −20 °C. The ZO essential oil was prepared just before being used by being diluted with 100% DMSO, resulting in a 1 g/mL solution.

### 4.2. Cell Culture

HeLa cells that were used for performing experiments were commercially obtained (ATCC, Manassas, VA, USA) and maintained in Dulbecco’s Modified Eagle’s Medium plus 10% fetal bovine serum (FBS) and antibiotics (100 U/mL penicillin and 100 µg/mL streptomycin). Cells were maintained in an incubator that maintained proper culture conditions (humidified at 37 °C with 5% CO_2_). HeLa cells were passed regularly. At the indicated times for specific experiments, culture media and cells were harvested for ELISA, flow cytometry, immunofluorescence assay, and Western blot analysis.

### 4.3. Flow Cytometry

Annexin-V-FITC/propidium iodide (PI) staining was applied to measure cancer cell apoptosis. HeLa cells were seeded at a density of 0.3 × 10^6^ cells/well in 3-cm dishes in complete media overnight. Cells were treated with ZO essential oil at 1:6000 and 1:3000 dilutions for 24 h. After treatment, HeLa cells were collected by trypsinization, washed with 1X PBS, resuspended in the binding buffer (50 mM Tris-HCl (pH 7.5), 5 mM EDTA, 0.5 mM DTT, and 50% glycerol), and stained for apoptotic cells using Alexa Fluor^®^ 488 annexin V (ImmunoTools, Deutschland, Germany) and PI (Sigma Aldrich, Saint Louis, MO, USA) for 15 min in the dark at room temperature (RT). Then, the analysis was performed by flow cytometry, and the results were recorded as the number of cells in each category.

### 4.4. Enzyme-Linked Immunosorbent Assay (ELISA)

HeLa cells were incubated with different dilutions (1:50,000–1:25,000) of ZO essential oil overnight (24 h), and the culture media were collected and subjected to ELISA to measure the amount of IL-6 (BioLegend, San Diego, CA, USA). The steps included coating the capture antibody overnight, blocking the plate, adding the sample culture media and the cytokine standard, adding the detection antibody, adding an avidin–horse radish peroxidase (HRP) solution, and adding a tetramethylbenzidine (TMB) substrate solution. The last step was the addition of a stop solution (2 N sulfuric acid). The measurements were done at 570 and 450 nm with an ELISA plate reader.

### 4.5. Western Blot Analysis

To investigate apoptosis signaling in HeLa cells after treatment with ZO essential oil, Western blot analysis was performed. Briefly, HeLa cells at a density of 0.05 × 10^6^ cells/well were seeded in 24-well plates and allowed to grow to confluence (0.2 × 10^6^ cells/well). Cells were incubated with ZO essential oil (various dilutions) for 24 h. Cell lysates were harvested using a 1X reducing Laemmli buffer that contained SDS and 2-mercaptoethanol (2-ME). SDS-PAGE was performed to separate proteins at different molecular weights, then the proteins were electroblotted onto polyvinylidene fluoride (PVDF) membranes. After blocking the membranes with 5% skim milk, they were incubated with specific antibodies (Cell Signaling Technology, Danvers, MA, USA): a rabbit anti-caspase-3 antibody, a rabbit anti-caspase-9 antibody, and a rabbit anti-PARP antibody. All of these three primary antibodies recognized the pro (full-length) molecules and the active (cleaved) molecules. Moreover, the primary antibodies included a mouse anti-actin antibody, a phosphospecific rabbit anti-phosphorylated ERK1/2 antibody, a mouse anti-total ERK1/2 antibody, a rabbit anti-phosphorylated Akt antibody, and a mouse anti-total Akt antibody. Next, membranes were washed with TBS-T and incubated with appropriate secondary antibodies (LI−COR Biosciences, Lincoln, NE, USA) for 2 h. The Western blot signals were captured by using an Odyssey^®^ CLx Imaging System (LI−COR Biosciences, USA).

### 4.6. Immunofluorescence Study

We performed immunofluorescence staining using a previously described method [11]. Briefly, the cells were cultured to grow on glass cover slips overnight and then treated with ZO essential oil for 24 h in a serum-free condition. Before harvesting cells, we stimulated the cells with 100 ng/mL of epidermal growth factor (EGF) for 15 min. Fixation was done for 15 min using 4% paraformaldehyde (for pAkt staining) or absolute methanol (for pERK1/2 staining). Following fixation with 4% paraformaldehyde, cells required permeabilization by using 0.3% TritonX-100 for 5 min. The cells were then blocked with 1% BSA in PBS for 1 h before being incubated with specific antibodies (a rabbit anti-phosphorylated ERK1/2 or rabbit anti-phosphorylated Akt antibody) at 4 °C overnight. The sample coverslips were incubated with appropriate secondary antibodies for 2 h in the dark. The nuclei of HeLa cell samples were stained with Hoechst 33342. Fluoromount-G (SouthernBiotech, Birmingham, AL, USA) was used to mount the sample cover slips, and the positive signal of pERK1/2 and pAkt was visualized and captured by a fluorescent microscope (microscope, AX70 Olympus, Shinjuku-ku, Tokyo, Japan).

### 4.7. Data and Statistical Analysis

All experiments were repeated at least three times, and the SPSS software (SPSS Inc., Chicago, IL, USA) was used for statistical analyses. Results are exhibited as mean ± standard deviation (SD). One-way ANOVA was performed, followed by Tukey’s post hoc multiple comparisons. *p* values that were less than 0.05 were considered statistically significant.

## 5. Conclusions

Our study discovered that the essential oil isolated from *Zingiber ottensii* induced the apoptosis of human cervical cancer cells. This event was regulated through the activation of caspases and PARP, which indicate an active intrinsic apoptotic pathway. Moreover, *Zingiber ottensii* essential oil potentially inhibited cellular proliferation and survival signaling via the suppression of ERK and Akt phosphorylation. Our findings support the potential use of ZO essential oil as an anti-cancer agent for treating patients with cervical cancer.

## Data Availability

All data, tables and figures are original.

## Supplementary Materials

The following are available online at https://www.mdpi.com/article/10.3390/plants10071419/s1, Figure S1: The effect of ZO essential oil on human primary fibroblast cell viability. The viability of human primary fibroblast cell viability upon treated with ZO essential oil for 24 h was tested by MTT assay. The treatment concentrations of ZO essential oil were prepared by performing a 2-fold dilution before adding into the cells. The experiment was performed three times (triplicate each time), Figure S2: The effect of ZO essential oil at the dilution of 1:6000 and 1:3000 on the morphology of human primary fibroblast cell. The morphological changes of human primary fibroblast cell were visualized by a phase-contrast microscope after treated with ZO essential oil at indicated dilutions for 24 h.
