# Supplementary material for: Essential Oil from Zingiber ottensii Induces Human Cervical Cancer Cell Apoptosis and Inhibits MAPK and PI3K/AKT Signaling Cascades
Source: Plants (Basel). 2021 Jul 12;10(7):1419. doi: 10.3390/plants10071419 (PMC8309419; doi:10.3390/plants10071419)
Supplement: Supplementary file 1 [file plants-10-01419-s001.zip › plants-1270794-supplementary.pdf]

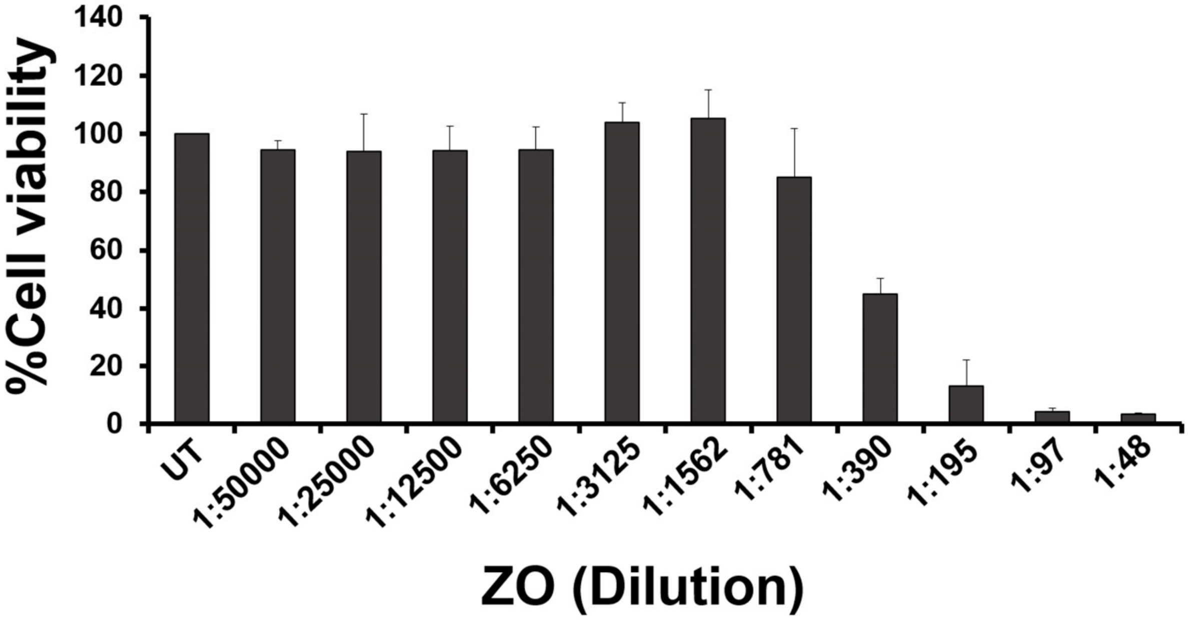

Figure S1. The effect of ZO essential oil on human primary fibroblast cell viability. The viability of human primary fibroblast cell viability upon treated with ZO essential oil for 24 h was tested by MTT assay. The Table 2. fold dilution before adding into the cells. The experiment was performed three times (triplicate each time).

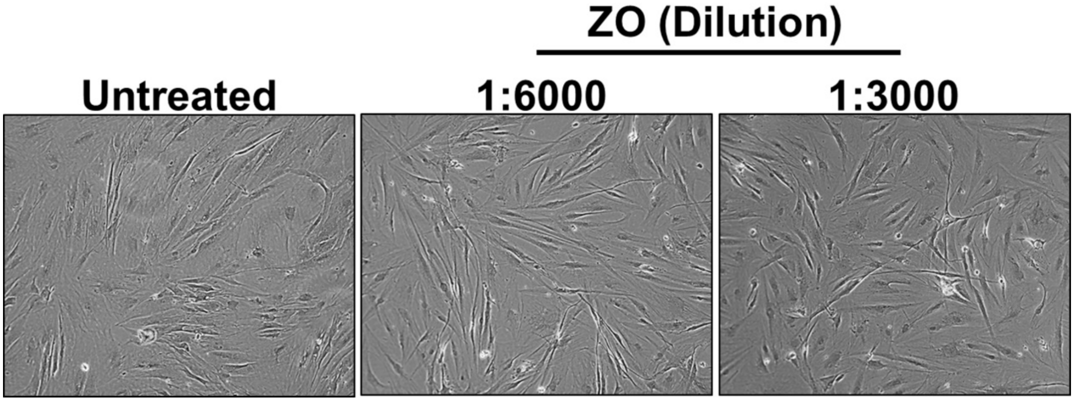

Figure S2. The effect of ZO essential oil at the dilution of 1:6000 and 1:3000 on the morphology of human primary fibroblast cell. The morphological changes of human primary fibroblast cell were visualized by a phase-contrast microscope after treated with ZO essential oil at indicated dilutions for 24 h.
